# The non-linear correlation between the volume of cerebral white matter lesions and incidence of bipolar disorder: A secondary analysis of data from a cross-sectional study

**DOI:** 10.3389/fpsyt.2023.1149663

**Published:** 2023-03-16

**Authors:** Hui Du, Bing Yang, Hui Wang, Yaqing Zeng, Jianpin Xin, Xiaoqiang Li

**Affiliations:** ^1^Department of Blood Transfusion, Affiliated Xiaolan Hospital, Southern Medical University, Zhongshan, Guangdong, China; ^2^Neurological Department and Stroke Center, the First Affiliated Hospital of Jinan University and Clinical Neuroscience Institute, Jinan University, Guangzhou, Guangdong, China; ^3^Department of Neurology, Affiliated Xiaolan Hospital, Southern Medical University, Zhongshan, Guangdong, China; ^4^Department of Radiology, Affiliated Xiaolan Hospital, Southern Medical University, Zhongshan, Guangdong, China

**Keywords:** cerebral white matter, bipolar disorder, secondary analysis, magnetic resonance imaging, non-linear correlation, cross-sectional study, dryad

## Abstract

Cerebral white matter lesions (WML) are major risk factors for bipolar disorder (BD). However, studies on the association between cerebral WML volume and BD risk are limited. This study aimed to investigate the relationship between cerebral WML volume and BD incidence. This is a secondary retrospective analysis of patients (*N* = 146, 72 males, 74 females, mean age = 41.77 years) who have previously undergone magnetic resonance imaging examinations. Information was obtained from the Dryad database. Univariate analysis, piecewise linear regression model, and multivariable logistic regression model were used for statistical analysis. A non-linear relationship was recognized between the cerebral WML volume and BD incidence, in which the inflection point of the WML volume was 6,200 mm^3^. The effect sizes and confidence intervals on the left and right sides of the emphasis point were 1.0009 (1.0003, 1.0015) and 0.9988 (0.9974, 1.0003), respectively. Subgroup analysis (WML volume < 6,200 mm^3^) showed that the cerebral WML volume (for 0.1 mm^3^ increase) was positively related to the BD incidence (OR = 1.11, 95% confidence interval [CI] (1.03, 1.21)). Here we show that the cerebral WML volume is positively and non-linearly correlated to the BD risk. Volumetric analysis of WML provide a better understanding of the association between WML and the BD risk, and thereby the pathophysiological mechanisms of BD.

**Graphical abstract:**

A non-linear relationship between the volume of cerebral white matter lesions (WML) and bipolar disorder (BD) incidence is shown. The cerebral WML volume is positively and non-linearly correlated to the BD risk. The correlation is stronger when the cerebral WML volume was <6,200 mm^3^.

**Graphical Abstract:**
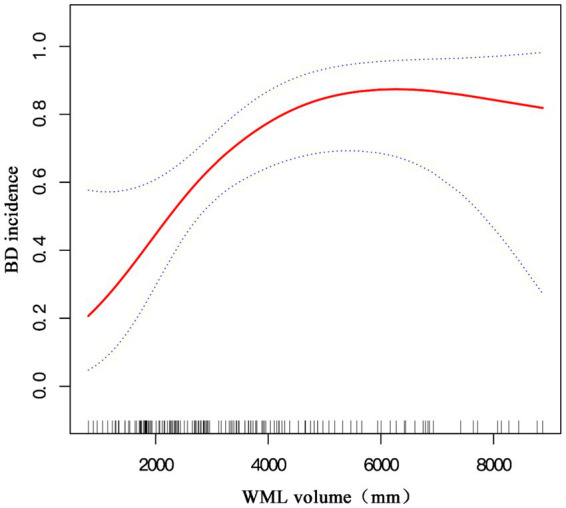
A non-linear relationship between the volume of cerebral white matter lesions and bipolar disorder incidence is shown after adjusting for age; sex; lithium, atypical antipsychotic, antiepileptic, and antidepressant drug use; BMI; migraine; smoking; hypertension; diabetes mellitus; substance and alcohol dependency; and anxiety disorder.

## 1. Introduction

Emotional fluctuations are very common in life. However, when mood swings are violent and persistent or lead to significant pain or damage, emotional disorders may be a potential cause. Bipolar disorder (BD) is a disease of high heritability characterized by repetitive episodes of elation and depression, combined with periods of normal mood in most cases (1, 2). BD is the sixth leading cause of disability worldwide and has a lifetime predominance of approximately 1–3% within the common population (3, 4).

The real causes of BD likely vary among people, and its precise underlying mechanism remains unclear (5). There are many studies on the causes and mechanisms of BD. Neuroimaging studies of bipolar disorder have revealed an association to abnormalities in the neural circuitry which regulate emotion and reward processing (6–8). The ventral system, which regulates emotional perception, includes brain structures such as the amygdala, the insula, the ventral striatum, the ventral anterior cingulate cortex, and the prefrontal cortex (9). The dorsal system, responsible for emotional regulation, includes the hippocampus, the dorsal anterior cingulate cortex, and other parts of the prefrontal cortex (9, 10). Phillips et al. (9) describe a model of BD that mood swings may occur when the ventral system is overactivated and the dorsal system is underactivated (9, 10). Studies have shown that mitochondrial impairment and oxidative stress may be involved in the development and progression of BD (11–13). A mate-analysis concluded that mitochondrial modulators have a significant antidepressant effect in BD patients (14). Another study showed that a deficiency in Glucose-6-phosphate dehydrogenase activity has been linked to bipolar disorder and has a positive relationship with mitochondrial impairment (15). Charles Okanda Nyatega’ findings imply that BD may be linked to striatal functional brain alterations and structural dysconnectivity (16). In addition, decreased levels of L-tryptophan, which causes increased pain sensitivity and cognitive impairment, were found in patients with BD (17).

As the mechanisms for BD remain unclear, and there are currently no validated biological markers, mental health professionals continue to rely on a phenomenology-based diagnostic system to diagnose BD (The Diagnostic and Statistical Manual of Mental Disorders, DSM). There is often a long period of inadequate or incorrect treatment before the official BD diagnosis is made (18). Therefore, challenges remain in identifying stable biomarkers for BD, to better understand the neurobiology and reach a more accurate diagnosis and treatment. The development of neuroimaging techniques, in particular the non-invasive measurement of brain structures from magnetic resonance imaging (MRI) scans, represents a unique approach to identify the brain structural variations associated with BD.

Neuroimaging studies have found differences in the volume of various brain regions between BD and healthy control (6). Accumulating evidence links BD to cerebral white matter lesions (WML) observed by MRI (19–22). Furthermore, WML is associated with poor clinical course of patients with BD (23). However, the association between BD and WML has not been clearly characterized, as WML at different regions of the central nervous system and detected with different methods have been used for this matter.

Volumetric analysis is common within the field of structural neuroimaging (24, 25). However, few relevant studies have assessed the relationship between cerebral WML volume and incidence of BD (26, 27). Here, we investigated the relationship between WML volume and BD risk in order to find a validated biological marker for BD and address the limitations of previous studies. This is a secondary analysis based on previously published data.

## 2. Methods

### 2.1. Data source

We used data published in the “Dryad” database.^1^ The database allows users to freely download raw data. Based on the Dryad Terms of Service, the exposed data of the paper can be used to re-analyze different scientific hypotheses (28, 29). We cite the Dryad dataset in the present study.

The database records included the following variables for secondary analysis: age; illness duration; WML volume; sex group; lithium, atypical antipsychotic, antiepileptic, and antidepressant drug use; body mass index (BMI); migraine; smoking; hypertension; diabetes mellitus; substance and alcohol dependency; and anxiety disorder.

### 2.2. Study design and population

The original research, BIPFAT study, was designed as a retrospective cohort study of 154 subjects (75 males, 79 females, mean age = 42.95 years) enrolled at the Medical University of Graz in Austria (26). The data used in this research can be downloaded from Dryad (Supplementary material) (30).

### 2.3. Participants

Inclusion Criteria: Patients that took part in the single-center BIPFAT study in Austria as inpatients or outpatients of the Medical University of Graz and were diagnosed of BD I or BD II according to the DSM-IV criteria. Participants were required to be in a euthymic state (Hamilton Rating Scale for Depression (HAM-D) and Young Mania Rating Scale (YMRS) scores: <11 and <9, respectively) and to have signed a written informed consent form. The ethics committee of the Medical University of Graz approved this study.

The exclusion criteria were the presence of systemic lupus erythematosus, rheumatoid arthritis, hemodialysis, inflammatory bowel disease, chronic obstructive pulmonary disease, and neurodegenerative and neuroinflammatory disorders (i.e., Alzheimer’s disease, Huntington’s disease, multiple sclerosis, and Parkinson’s disease). More details on the study exclusion criteria are detailed in the original study (26). An additional exclusion criterion for this study was the presence of a lifelong psychiatric diagnosis. Eight participants with WML larger than 9,000 mm^3^ were excluded (31, 32), and the remaining 146 participants entered the final analysis.

### 2.4. Data collection and measurements

The original study database included demographic parameters, complete actual and lifetime psychiatric history using the Structured Clinical Interview for DSM Disorders (SCID), anthropometric measures, medication history, fasting blood, blood pressure, electroencephalogram (EEG), different lifestyle questionnaires, and MRI of the cerebral cortex. All participants were former inpatients or outpatients of the Medical University of Graz and had a diagnosis of BD based on the DSM-IV criteria.

### 2.5. Statistical analysis

Continuous variables were presented as mean ± standard deviation. Categorical variables were expressed as frequencies and percentages. Continuous variables were compared using the two-sample *t*-test or Wilcoxon rank-sum, and categorical variables were compared using the *χ*^2^ test and Fisher’s exact test. The generalized additive model (GAM) was used to confirm a non-linear relationship between the cerebral WML volume and BD incidence. Next, the saturation effect of cerebral WML volume on BD was calculated based on smoothing curves using a two-stage logistic regression model. Univariate and multivariate Cox proportional risk models were used to assess the relationship between the cerebral WML volume and BD risk. We used three models: model 1 (crude model), model 2 (adjusted for age and sex), and model 3 (adjusted for age; sex; lithium, atypical antipsychotic, antiepileptic, and antidepressant drug use; BMI; migraine; smoking; hypertension; diabetes mellitus; substance and alcohol dependency; and anxiety disorder). Differences were considered statistically significant when the calculated *p*-value was <0.05. All analyses were performed using the statistical software R (https://www.R-project.Org, The R Foundation) and EmpowerStats (http://www.empowerstats.com, X&Y Solutions Inc., Boston, MA, United States) (33–35).

## 3. Results

### 3.1. Characteristics of study participants

Overall, 74 women (50.68%) and 72 men (49.32%) were retrospectively analyzed. The baseline characteristics of the patients with BD (BD group) and healthy participants (control group) are shown in Table 1. The mean patient age was 41.77 years (SD, 13.81). Overall, the average WML volume was 3499.24 mm^3^. The BD group had a higher mean WML volume (3948.86 mm^3^) compared to the control group (2686.30 mm^3^; *p* < 0.01).

**Table 1:** Baseline characteristics of participants with or without Bipolar Disorder (*N* = 146).

Variables	Total	Bipolar disorder	Healthy controls	*p*-value
No. of participants	146	94	52	
Age (years)	41.77 ± 13.81	42.91 ± 13.05	39.70 ± 15.00	0.179
Sex (male)	72 (49.32%)	50 (53.19%)	22 (42.31%)	0.208
BMI (kg/m^2^)	26.80 ± 4.86	27.89 ± 4.66	24.38 ± 4.62	<0.001
WML volume(mm^3^)	3499.24 ± 1903.22	3948.86 ± 1841.33	2686.30 ± 1751.93	<0.001
Illness duration (years)	/	17.74 ± 11.84	/	
Depression	/	12.93 ± 12.25	/	
Mania	/	8.36 ± 8.88	/	
Age First Episode (years)	/	24.83 ± 10.58	/	
Lithium (*n*, %)	16 (10.96%)	16 (17.02%)	0 (0.00%)	0.002
Antiepileptic (*n*, %)	17 (11.64%)	17 (18.09%)	0 (0.00%)	0.001
Atypical Antipsychotic (*n*, %)	39 (26.71%)	39 (41.49%)	0 (0.00%)	<0.001
Antidepressive drug (*n*, %)	34 (23.29%)	34 (36.17%)	0 (0.00%)	<0.001
Migraine (*n*, %)	37 (25.52%)	22 (23.40%)	15 (29.41%)	0.428
Smoking (*n*, %)	61 (41.78%)	48 (51.06%)	13 (25.00%)	0.002
Hypertension (*n*, %)	37 (25.34%)	26 (27.66%)	11 (21.15%)	0.387
DM (*n*, %)	7 (4.79%)	7 (7.45%)	0 (0.00%)	0.044
Substance dependency (*n*, %)	11 (7.53%)	11 (11.70%)	0 (0.00%)	0.010
Alcohol dependency (*n*, %)	17 (11.64%)	17 (18.09%)	0 (0.00%)	0.001
Anxiety disorder (*n*, %)	22 (15.17%)	22 (23.66%)	0 (0.00%)	<0.001

Abbreviations: BMI, body mass index; DM, diabetes mellitus; WML were normalized by individual total intracranial volume for the calculations.

### 3.2. Non-linear relationship between WML volume and BD incidence

We aimed to characterize the actual relationship between WML volume and BD incidence. As WML volume is a continuous variable, the GAM was used to identify a non-linear relationship between the two variables. We found that this relationship was non-linear after adjusting for sex; age; lithium, atypical antipsychotic, antiepileptic, and antidepressant drug use; BMI; migraine; smoking; hypertension; diabetes mellitus; substance and alcohol dependency; and anxiety disorder. We used a two-piecewise linear regression model to calculate the inflection point of the WML volume, which was found to be 6,200 (log-rank test, *p* < 0.05; Table 2). A positive relationship between the WML volume and BD incidence was observed on the left side of the inflection point (OR: 1.0009, 95% CI: 1.0003, 1.0015, *p* < 0.01), whereas saturation was observed toward the right of the inflection point (OR: 0.9988, 95% CI: 0.9974, 1.0003, *p* = 0.1217; Table 2). Thus, to analyze the positive relationship between the WML volume and BD incidence, we selected patients with WML volume < 6,200 mm^3^.

**Table 2:** Results of two-piecewise linear regression model.

	Incidence of BD (OR, 95%CI)	*p*-value
Fitting model by standard linear regression	1.0004 (1.0000, 1.0008)	0.0382
Fitting model by two-stage linear regression		
The inflection point of WML volume(mm^3^)	6,200	
<6,200	1.0009 (1.0003, 1.0015)	0.0048
>6,200	0.9988 (0.9974, 1.0003)	0.1217
*p* for log likelihood ratio test	0.025	

We adjusted age; sex; lithium; atypical antipsychotic; antiepileptic; antidepressive drug; BMI; migraine; smoking; hypertension; diabetes mellitus; substance dependency; alcohol dependency; anxiety disorder. CI: confidence interval; BD: bipolar disorder.

### 3.3. Univariate analysis for BD incidence (WML volume < 6,200 mm^3^)

In order to address which factors are related to BD incidence, we performed univariate analysis (Table 3). Using the univariate Cox proportional risk model, we found that WML volume (OR = 1.10, 95% CI: 1.05, 1.14, per 0.1cm^3^), BMI (OR = 1.16, 95% CI: 1.06, 1.28), and smoking (OR = 3.86, 95% CI: 1.75, 8.49) were positively correlated with BD, whereas age, sex, migraine, and hypertension were not associated with BD.

**Table 3:** Results of univariate analysis between white matter lesions volume (<6,200 mm^3^) and bipolar disorder risk.

	Statistics	OR (95% CI)	*p*-value
WML volume (0.1 cm^3^)	29.50 ± 12.55	1.10 (1.05, 1.14)	<0.0001
Age	39.90 ± 13.09	1.02 (0.99, 1.05)	0.2012
BMI	26.69 ± 4.85	1.16 (1.06, 1.28)	0.0016
Sex			
Male	61 (47.66%)	1.0	
Female	67 (52.34%)	0.78 (0.38, 1.60)	0.4934
Migraine			
No	95 (74.80%)	1.0	
Yes	32 (25.20%)	0.81 (0.36, 1.85)	0.6244
Smoking			
No	71 (55.47%)	1.0	
Yes	57 (44.53%)	3.86 (1.75, 8.49)	0.0008
Hypertension			
No	98 (76.56%)	1.0	
Yes	30 (23.44%)	1.05 (0.45, 2.45)	0.9142

### 3.4. Multivariate analysis between WML volume and BD incidence (WML volume < 6,200 mm^3^)

To explore the relationship between WML volume and BD incidence, we used WML volume (0.1 mm^3^) as the independent variable, BD risk as the dependent variable, and age; sex; lithium, atypical antipsychotic, antiepileptic, and antidepressant drug use; BMI; migraine; smoking; hypertension; diabetes mellitus; substance and alcohol dependency; and anxiety disorder were adjusted for multivariate regression analysis.

In model 1, the WML volume was positively correlated with BD risk (odds ratio (OR) = 1.10, 95% CI: 1.05, 1.14, *p* < 0.001; Table 4), and the same positive correlation was observed in the minimum adjusting model (model 2: OR: 1.10, 95% CI: 1.05, 1.15, *p* < 0.0001). In model 3, the incidence of BD increased by 1.11 times per 0.1 mm^3^ increase in WML (OR = 1.11, 95% CI: 1.03, 1.21, *p* < 0.001).

**Table 4:** Relationship between white matter lesions volume and bipolar disorder risk in different models.

Variable	Crude model (OR,95% CI, *p*)	Model I (OR,95% CI, *p*)	Model II (OR,95% CI, *p*)
WML volume (for 0.1 cm^3^ increase)	1.10 (1.05, 1.14) <0.0001	1.10 (1.05, 1.15) <0.0001	1.11 (1.03, 1.21) 0.0067

Crude model was not adjusted for other variables; Model I was adjusted for age; sex; Model II was adjusted for age; sex; lithium; atypical antipsychotic; antiepileptic; antidepressive drug; BMI; migraine; smoking; hypertension; diabetes mellitus; substance dependency; alcohol dependency and anxiety disorder; Abbreviations: CI, confidence; OR, odds ratios; *p*, *p*-value.

## 4. Discussion

Our study shows a significant association between cerebral WML volume and BD risk, and this relationship is independent of other risk factors (OR: 1.11, 95% CI: 1.03, 1.21, *p* < 0.001 for 0.1 mm^3^ WML increase). Other studies have reported similar results (27, 36, 37). Our study not only evaluated the independent impact of cerebral WML volume and BD risk but also explored the non-linear relationship between them. The inflection point of the cerebral WML volume was calculated to be 6,200 mm^3^. When the inflection point was <6,200 mm^3^, the cerebral WML volume was positively correlated with the BD occurrence (OR: 1.0009, 95% CI: 1.0003, 1.0015, p < 0.001), indicating that patients with larger WML volume have a higher risk of BD. When the inflection point was >6,200 mm^3^, no positive relationship was found between the cerebral WML volume and BD risk (OR: 0.99988, 95% CI: 0.9974, 1.0003, *p* = 0.1217).

The measurements of WML are different. Some studies indicate that patients with BD are likely more vulnerable (almost twice) to developing WML changes than healthy participants (38). However, the degree of white matter and paraventricular hyperintensities was quantified according to the scoring method described by Coffey et al. (39) and was grouped into two categories (mild and more extensive) (38). Another study found that adolescent patients with BD had significantly increased numbers of WML compared with healthy individuals (40). However, WML incidence was evaluated on a four-point ordinal scale (none, mild, moderate, and severe) (40). The WML measurements were based on a qualitative rating scale, and the standards were confusing. In this study, 3 T MRI was performed (26), while some previous studies primarily report use of the 1.5 T MRI (41–44). 3T MRI is more sensitive to WML than 1.5 T MRI, which means our study may have found more WML than that found with 1.5 T MRI (45, 46).

Volumetric analysis has rarely been applied to the study of WML in BD (23, 27, 47), although this approach is common in structural neuroimaging. Through voxel-based morphometry for T1-weighted images (MRI), some researchers found that patients with BD have a greater cluster size than healthy people (37). Another study found that the WML volume of male patients with BD is closely related to the number of manic episodes (26). Also, WML volume seems to be correlated with familiarity and type of BP (27). However, we found a non-linear relationship between cerebral WML volume and BD incidence when the volumetric analysis was performed.

White matter may play an important role in the neurobiology of BD (48). One study found changes to bilateral white matter connectivity (i.e., decreased fractional anisotropy) during emotion regulation and sensory processing in participants with BD when compared with healthy controls (49). Global abnormalities in white matter tracts (seen by MRI) might account for the characteristic mood lability of BD (50). McDonald’s study showed that white matter reduction in the left frontal and temporoparietal regions was associated with the genetic risk of bipolar disorder (51). Karlsgodt et al. found that the white matter microstructural disruptions were predictive of functional outcomes in youth at high risk of developing psychosis (52). Lower fractional anisotropy, which reflects the collinearity and/or integrity of the fibers, has been consistently reported in white matter tracts involved in emotional processing and regulation in youth and adults with BD (53–56).

Higher incidence rates of BD are also associated with more abnormalities in white matter tracts (21). However, the presence of some factors such as obesity, metabolic syndrome, and cardiovascular risk factors, such as hypertension, diabetes, and age are associated with cerebral white matter lesions (57–59). In our study, age, hypertension, and diabetes were adjusted for in the multivariate regression analysis, and we found that the BD incidence did not increase when the cerebral WML volume increased to >6,300 mm^3^. We speculate that other white matter lesions-related factors (such as cerebral infarction, vasculitis, inflammation, and multiple sclerosis) are responsible for the increase in cerebral WML volume after a certain volume limit, showing no association with BD occurrence.

This study had several limitations. First, from a statistical point of view, the number of patients of the present study is insufficient. However, the study population included in this study was adequate from the perspective of studies on WML volume and BD, as most studies on BD collect data from less than 100 participants (23, 60–64). Therefore, future studies should further verify our results by expanding the sample size. Second, the literature suggests that different parts of white matter degeneration (deep or periventricular WML) affect the occurrence of BD. However, in our study, the WML volume does not distinguish the location of white matter degeneration, and further research is required to clarify this. Finally, our study was retrospective, cross-sectional and showed a non-linear association between WML volume and BD risk. Therefore, prospective basic and clinical studies are required to confirm this causal relationship.

Concerning the future directions, our study was only focused on the most used T2 MRI, thus future studies should attempt to perform resting state functional MRI (RS-fMRI) and diffusion tensor imaging (DTI), which could help in obtaining more detailed information about altered functional connectivity in brain areas that are impaired in BD.

In conclusion, this cross-sectional study demonstrated a non-linear relationship between WML volume and BD risk. Moreover, the relationship between WML volume and BD risk was positive when the WML volume was <6,200 mm^3^. In contrast, no positive relationship was observed between WML volume and BD risk when the WML volume was >6,200 mm^3^. Neuroimaging and subsequent volumetric analysis of WML led to a better understanding of the association between WML and the BD risk, which, in turn, provide further insights into the pathophysiological mechanisms of BD. However, larger sample size studies should further verify our results to confirm WML volume as a stable biomarker of BD incidence.

## Data availability statement

The original contributions presented in the study are included in the article/Supplementary material, further inquiries can be directed to the corresponding author.

## Ethics statement

Written informed consent was obtained from the individual(s) for the publication of any potentially identifiable images or data included in this article.

## Author contributions

XL and HD contributed to conception and design of the study and wrote the first draft of the manuscript. BY and YZ organized the database. JX and HW performed the statistical analysis. All authors contributed to the article and approved the submitted version.

## Conflict of interest

The authors declare that the research was conducted in the absence of any commercial or financial relationships that could be construed as a potential conflict of interest.

## Publisher’s note

All claims expressed in this article are solely those of the authors and do not necessarily represent those of their affiliated organizations, or those of the publisher, the editors and the reviewers. Any product that may be evaluated in this article, or claim that may be made by its manufacturer, is not guaranteed or endorsed by the publisher.

## Footnotes

^1^ https://datadryad.org/

## Abbreviations

WML: Cerebral white matter lesions
BD: bipolar disorder
MRI: magnetic resonance imaging
BMI: body mass index
GAM: generalized additive model
BIPFAT study, original project name: Fettstoffwechselstörungen und anthropometrische Besonderheiten bei PatientInnen mit bipolarer affektiver Störung
